# A Transportable Atomic Gravimeter with Constraint-Structured Active Vibration Isolation

**DOI:** 10.3390/s24082395

**Published:** 2024-04-09

**Authors:** Chuanjing Ruan, Wei Zhuang, Jiamin Yao, Yang Zhao, Zenghan Ma, Cong Yi, Qin Tian, Shuqing Wu, Fang Fang, Yinghong Wen

**Affiliations:** 1 National Institute of Metrology, Beijing 100029, China; 2 School of Automation and Intelligence, Beijing Jiaotong University, Beijing 100044, China; 3 Key Laboratory of State Administration for Market Regulation (Time Frequency and Gravity Primary Standard), Beijing 100029, China; 4 College of Instrumentation and Electrical Engineering, Jilin University, Changchun 130061, China

**Keywords:** atomic gravimeter, active vibration isolation, vibration coupling, noises analysis

## Abstract

Many efforts have been taken in recent years to push atomic gravimeters toward practical applications. We demonstrate an atomic gravimeter named NIM-AGRb2 that is transportable and suitable for high-precision gravity measurements. Constraint-structured active vibration isolation (CS-AVI) is used to reduce the ground vibration noise. The constraint structure in CS-AVI ensures that the isolation platform only has vertical translation, with all other degrees of freedom (DoFs) being constrained. Therefore, the stability of active vibration isolation is enhanced. With the implementation of CS-AVI, the sensitivity of NIM-AGRb2 reached as low as 20.5 μGal/Hz^1/2^. The short-term sensitivity could be further reduced to 10.8 μGal/Hz^1/2^ in a seismologic observation station. Moreover, we evaluated the system noise of the gravimeter, and the results were consistent with our observations.

## 1. Introduction

Absolute gravimetry with a precision at the micro-Gal (1 μGal = 10 nm/s^2^) level has been demonstrated [1,2,3,4,5,6,7,8] through gravimeters based on light-pulse atom interferometers [9]. In a quiet environment, atomic gravimeters have shown unprecedented sensitivity [1,2,3,4]. Additionally, the inherent advantages of atomic gravimeters, including their high repetition rate, long service life, and stability against drift, have naturally led to substantial efforts in advancing their application in field settings [10,11,12,13,14,15] or on mobile platforms [16,17,18,19,20,21,22,23,24]. In recent years, atomic gravimeters have been used in geophysical surveys, such as detecting volcano-related underground mass changes [25] and providing absolute gravity references for relative gravimeters [26]. These applications highlight the promising future of atomic gravimeters.

In field applications, vibration noise often constitutes the primary noise source for atomic gravimeters. Many vibration reduction techniques have been extensively discussed [27,28,29], which could be applied within the vibration isolation systems of atomic gravimeters. Active vibration isolation, based on commercial passive isolation platforms [30,31,32,33,34,35], is a compact and effective method for eliminating vibration noise. Atomic gravimeters are sensitive to low-frequency vibration noise. The resonance frequency of commercial passive vibration isolation platforms is typically around 0.5 Hz. Active vibration isolation can further reduce the resonance frequency to the level of 10^−2^ Hz, effectively mitigating the impact of low-frequency vibration noise above that on atomic gravimeters. However, active vibration isolation in field applications may encounter challenges, primarily due to the six degrees of freedom (DoFs) in passive isolation platforms, which can potentially lead to coupling between the horizontal and vertical vibrations [30,36] as well as inclination drift [35].

Many efforts have been devoted to enhancing the environmental adaptability of active vibration isolation. Three-dimensional active vibration isolation [33,34] can reduce the coupling effect of horizontal and vertical vibrations. Active tilt control in active vibration isolation [35] can effectively solve the tilt drift of isolation platforms.

Contrary to the complex active control approach, simplifying the structure could be an alternative strategy to increase the stability of active vibration isolation. In this work, we employ a constraint structure, which constrains the DoFs of a passive isolation platform, leaving only vertical translation freedom. Consequently, active isolation is only required in the vertical direction, and the effects of vibration coupling and tilt drift are limited. Constraint-structured active vibration isolation (CS-AVI) is simple, obviating the need for precise matching and critical adjustments, making it well suited for field applications involving atomic gravimeters.

Our newly developed transportable atomic gravimeter named NIM-AGRb2 employs CS-AVI to reduce the vibration noise. The system volume of the gravimeter is more compact compared to our previous work [5], which makes it suitable for transportable high-precision gravity measurements. With the implementation of CS-AVI, the sensitivity of NIM-AGRb2 reaches 20.5 μGal/Hz^1/2^ in a laboratory and 10.8 μGal/Hz^1/2^ during a transportation test. To further optimize the system, the system noise is analyzed in detail.

## 2. Apparatus

### 2.1. Experiment Setup

The physical package of NIM-AGRb2 is a cylinder with a diameter of 50 cm and a height of 80 cm. The height for the atomic interference range is over 20 cm, while maintaining the potentiality for high-precision gravity measurement in a transportable atomic gravimeter. The laser system consists of two lasers, with the main laser being a fiber laser and the slave laser being a diode laser. The frequency of the main laser is locked on the $|5S_{1/2},F=2\rangle \to |5P_{3/2},F^{\prime }=3\rangle$ transition of ^87^Rb atoms with modulation transfer spectroscopy. Through an optical phase-locked loop (OPLL), the slave laser is phase-locked to the main laser with a frequency offset of 6.834 GHz. All optomechanical components are integrated into two compact aluminum boxes with dimensions of 470 × 470 × 100 mm^3^ and 320 × 320 × 100 mm^3^, respectively.

**Figure 1:**
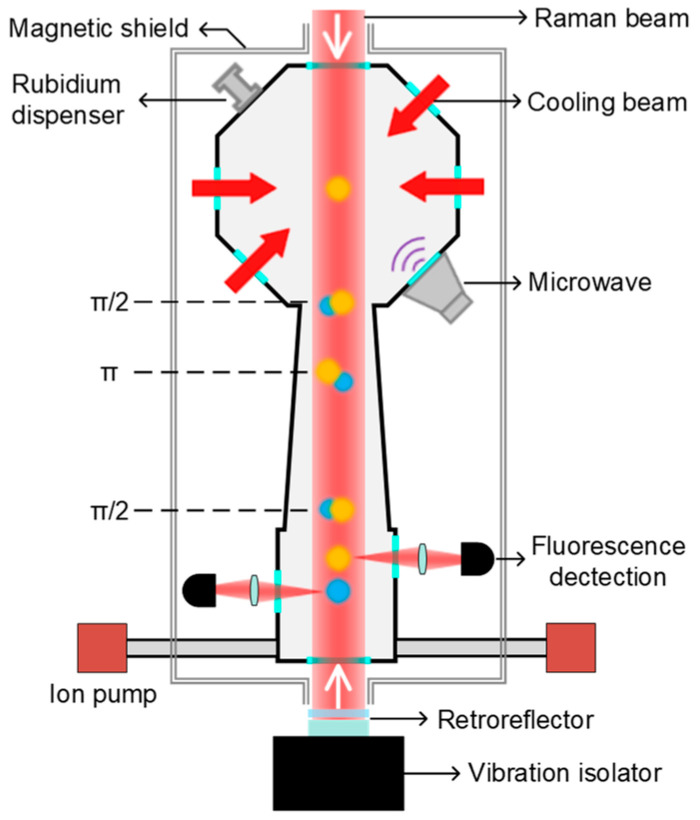
Schematic experimental setup of the atomic gravimeter. The top yellow sphere represents the ^87^Rb atoms that are trapped and cooled to 2 μK in the ground state |F=2〉 using a three-dimensional magneto-optical trap. Then, the atoms fall freely and are sequentially subjected to state preparation and atomic interference operations. Fluorescence detection is used to detect the final state atoms of |F=1〉 (blue sphere) and |F=2〉.

Figure 1 shows the schematic structure of the gravity measurement setup. Initially, ^87^Rb atoms are cooled and trapped using six cooling laser beams (two additional beams not shown). Approximately 10^8^ atoms are loaded in a three-dimensional magneto-optical trap (3D-MOT) within 500 ms. Subsequently, the atomic cloud is further cooled to 2 μK through a polarization gradient cooling process. After turning off the cooling beams, the cold atomic cloud starts to fall freely. By applying microwave and Raman optical selection processes, the atoms are prepared in the magnetically insensitive state $|F=1,m_{F}=0\rangle$. After the selection process, the number of atoms still remains about 5 × 10^6^, and the atomic cloud is further cooled down to 200 nK in the vertical direction. Subsequently, a π/2-π-π/2 Raman pulse sequence is applied to the atoms to realize atomic interference. We finely adjust the bias magnetic field so that the free-evolution time *T* can reach 105 ms. Using the method of normalized fluorescence detection, we obtain the transition probability *P*, $P=[1-C\cdot \cos(k_{eff}gT^{2}-\alpha T^{2})]/2$, where *C* is the fringe contrast, *k_eff_* is the effective wavevector of the Raman beams, *g* is the gravity acceleration, and α is the chirp rate of the Raman beams used to compensate for the Doppler shift during the atoms’ free fall. In our experiment, one measurement cycle was 1 s.

### 2.2. Constraint-Structured Active Vibration Isolation

**Figure 2:**
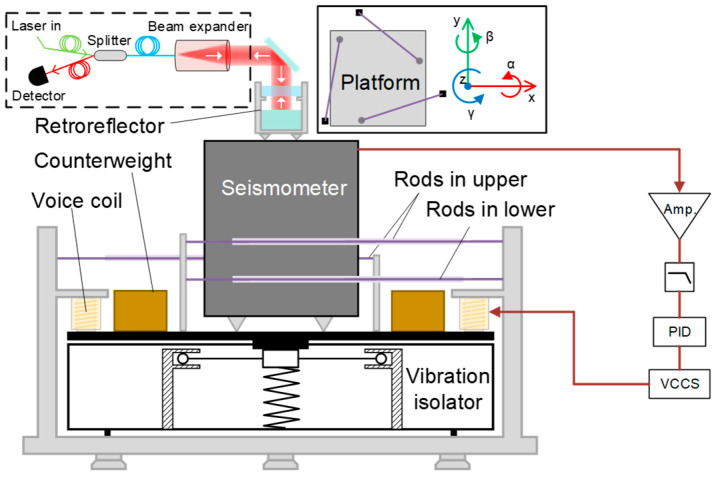
Structure of the CS-AVI process. The solid framework is the top view of the passive vibration isolator and rods. The dashed framework represents the scheme to monitor the tilt of the vibration isolator.

CS-AVI is placed under the physical package to reduce the vibration noise. The structure of the active isolator is shown in Figure 2. The isolation system is based on a commercial passive isolator (Minus K 25-BM10, Los Angeles, CA, USA). The passive vibration isolation employs a negative-stiffness mechanism [37] to achieve a low resonant frequency of 0.5 Hz, which can attenuate vibration noise above 1 Hz. Counterweights are used to adjust the levelness of the platform of the passive isolator. A constraint mechanism with five steel rods (introduced in the next paragraph) is used to limit the DoFs of the passive isolation platform. A seismometer (Geolight GL-CS60, Somerset, UK) is installed on the passive isolation. The residual vibration noise of passive isolation is sampled with the seismometer. The signal of the seismometer is amplified and then filtered through a low-pass filter to remove high-frequency noise. The signal is then sent into a proportional–integral–derivative (PID) circuit. The PID circuit outputs a voltage signal to control a voltage-controlled current source (VCCS), which outputs feedback current to two voice coils. These coils exert feedback force to the passive isolation platform to reduce the residual vibration noise.

Five steel rods are employed [38], with three rods on the upper layer (three rods are coplanar) and two rods on the lower layer (two rods are coplanar), and the two surfaces of the upper and lower layers are parallel. The solid frame in Figure 2 shows the top view of the five rods. One end of the rod is connected to a fixed column (black squares) and cannot be moved, while the other end is attached to the column (grey dots) on the passive isolation platform and can be moved with the platform. The three upper rods restrict the degrees of freedom (DoFs) of x, y, and γ, as motion in these three freedoms would necessitate at least one rod to be stretched or compressed. Since metal rods are difficult to stretch or compress, such deformation effectively suppresses the motion of the passive platform in these DoFs. Under the combined action of all five rods, the DoFs of α and β are limited, as these two freedoms would cause the rectangle formed by the upper and lower wires to become a trapezoid, leading to rod compression. This also suppresses the impact of external vibrations on these two degrees of freedom. However, this mechanical structure cannot restrict the z-direction freedom, as displacement in this direction is perpendicular to the rods, with negligible damping by the rods over a small range. Therefore, this structure can achieve restriction in five degrees of freedom. With this structure, we only need to actively isolate vibration noise in the vertical direction. In practice, we can just use two spirit levels to simply adjust the level of the passive vibration isolation platform, which makes the isolator easy to set up.

**Figure 3:**
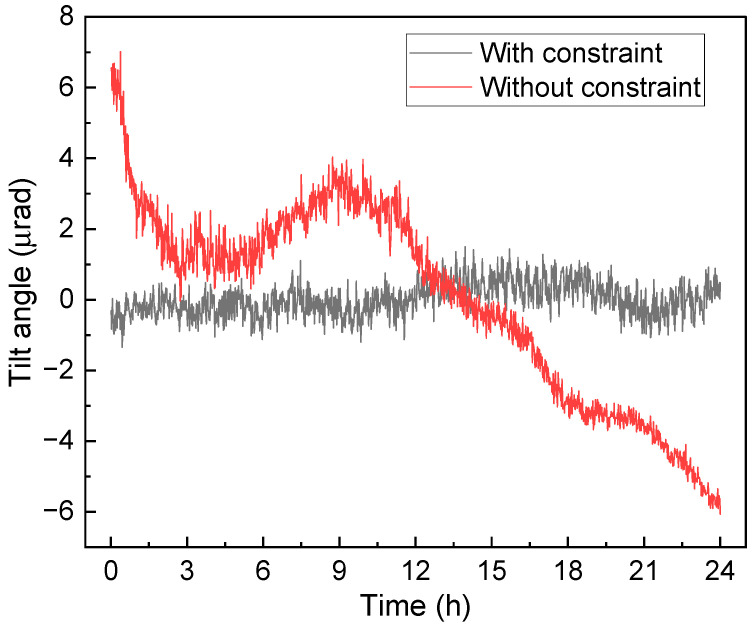
Tilt drift of the passive isolation platform.

The tilt drift of the passive isolation platform is monitored to verify the performance of the constraint structure, as shown in the dashed framework in Figure 2. A laser beam is coupled into the fiber splitter via dual-port coupling and directed towards the retroreflector through a beam expander to form a spatial laser beam. The retroreflector can be adjusted to couple the reflected beam into the fiber splitter and detected with a detector. The stability of the isolation platform can be reflected by the signal from the detector. The experimental results are shown in Figure 3. The red curve demonstrates that, without the constraint structure, the vibration isolation platform consistently exhibits tilt drift. This drift may be attributed to the rough leveling adjustment of the platform using two spirit levels, along with slight force imbalance between the two voice coils. Additionally, vibrations and airflow might also contribute to the drift of the isolation platform. The black curve indicates that the constraint structure is capable of suppressing the tilt drift of the vibration isolation platform to within ±1 μrad, significantly enhancing the stability of the active vibration isolation. Therefore, this limiting structure effectively restricts the DoFs of the system. The performance of vibration attenuation will be discussed in detail in the Section 4.

## 3. Gravity Measurement

### 3.1. Laboratory Results

**Figure 4:**
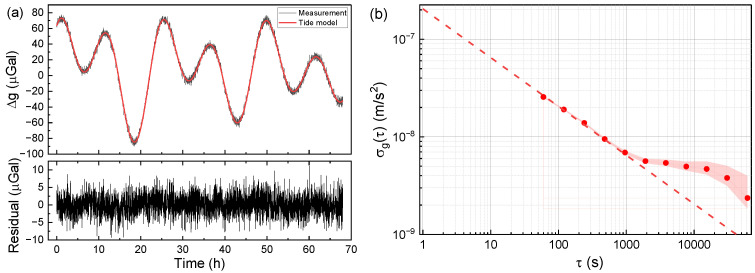
Gravity measurement results from the laboratory. (**a**) The measured gravity data and the residuals of gravity subtracted from the tide model. (**b**) The Allan standard deviation of the residuals in (**a**). The filled region indicates the confidence intervals of the points. The red dashed line corresponds to a sensitivity of 20.5 μGal/Hz^1/2^.

Figure 4a displays the 68 h continuous measurement obtained in a laboratory at the Changping campus of the National Institute of Metrology (NIM) in Beijing. In the top diagram, the black line denotes the gravity tide curve measured with the gravimeter NIM-AGRb2, with each data point representing a 60 s average. The red line represents the theoretical tide model. The bottom graph shows the residuals of the measured data after the correction of the theoretical tides. The results exhibit a good agreement between the measured data and the theoretical tides. Subsequently, the residuals were evaluated using Allan deviation, and these results are presented in Figure 4b. The fitting results reveal that the sensitivity of the gravimeter is 20.5 μGal/Hz^1/2^, and its resolution can reach up to 0.69 μGal after an integration time of 1000 s. However, the Allan standard deviation after 1000 s departs from the expected decreasing trend of *τ*^−1/2^. This deviation may potentially arise from the temperature variation in the laboratory and the influence of the air flow generated by the air conditioner.

### 3.2. Transportation Test

**Figure 5:**
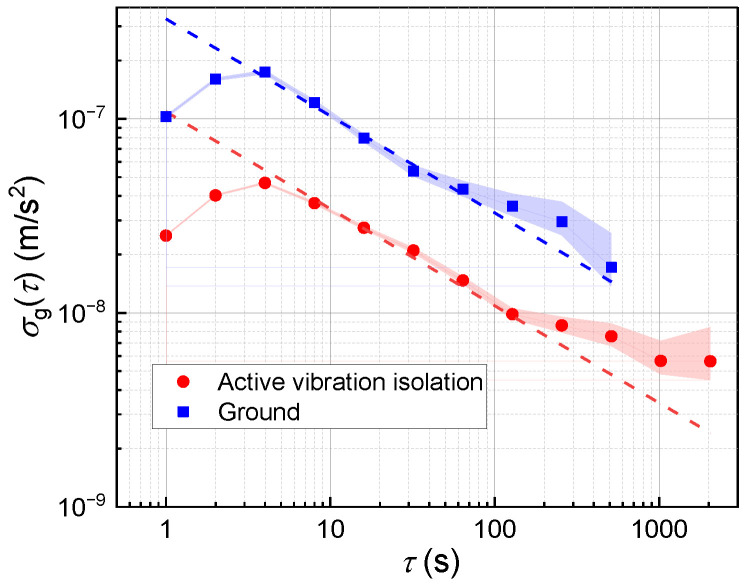
Allan standard deviation of gravity data in the seismic station. The filled region indicates the confidence intervals of the points. The blue and red dashed lines indicate short-term sensitivities of 33.1 μGal/Hz^1/2^ and 10.8 μGal/Hz^1/2^ under the two conditions in the legend, respectively.

To verify the transportability of the gravimeter NIM-AGRb2, the system was transported to a seismic station where the vibration noise was lower than that in our laboratory, through which the noise floor of the instrument after transportation can be verified. The Allan standard deviation results from the seismic station are displayed in Figure 5. Note that the measured gravity data for computing Allan standard deviation is not a 60 s average for each data point, and the initial bump of two Allan standard deviation points is due to an integrator locking the measured gravity acceleration onto the tidal change [39].

The blue squares represent the Allan standard deviation results obtained in directly underground vibration conditions without any vibration isolations in the seismic station. The blue fitted line indicates a sensitivity of 33.1 μGal/Hz^1/2^, denoting that the vibration noise level in the chamber was sufficiently low for our system to achieve micro-Gal measurements, even without any vibration isolations. Moreover, when employing active vibration isolation, we achieved a further measurement sensitivity of 10.8 μGal/Hz^1/2^, as illustrated by the red dashed line in Figure 5. This observation confirms the effectiveness of CS-AVI even in environments with low vibration levels. The instrument exhibits lower noise levels than those observed in the laboratory environment, indicating that the system has no performance degradation due to transport bumps and is capable of transportable high-precision gravity measurements.

## 4. Analysis for System Noises

The sensitivity is related to the system noise. In order to improve the measurement sensitivity, we performed a detailed analysis of the system noise. Atomic interference fringes directly reflect the noise level of the system. The noises of interference fringes can be classified into two types: fringe amplitude noise and phase noise. The fringe amplitude noise refers to the random fluctuations observed in the amplitude of each fringe measurement point. On the other hand, the fringe phase noise represents the random phase shifts of the interference fringe. In our evaluation, we consider the amplitude noise as a whole. Additionally, we analyze the Raman laser phase noise and vibrational noise with regard to the fringe phase noise.

### 4.1. Fringe Amplitude Noise

**Figure 6:**
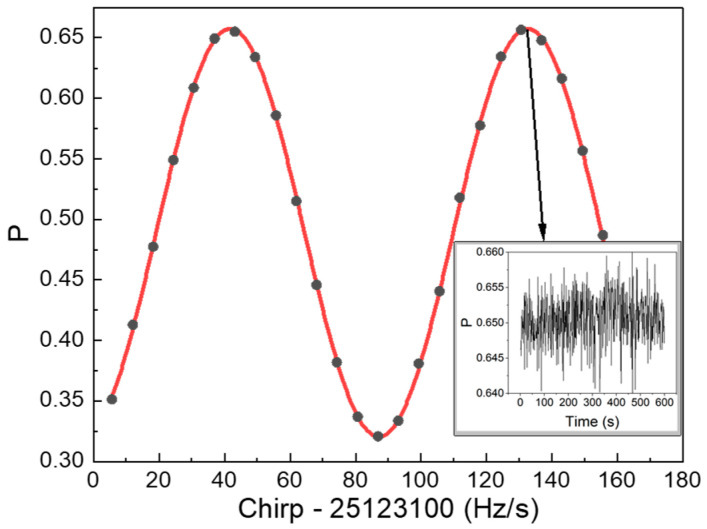
Interference fringe of the atomic gravimeter for T = 105 ms. Each black dot corresponds to the transition probability obtained from a single measurement with a 1 s interval. The red curve represents the sinusoidal fitting of the fringe. The inset graph shows the results of sampling at the peak of the fringe for 600 s.

In our system, a horizontal detection scheme was employed for detection [36]. The atomic interference fringes are shown in Figure 6, with the black dots showing the experimental results, and the red line showing the sinusoidal fit curve to the measured data. The data sampled at the peak of the fringe (as shown in the inset graph in Figure 6) were utilized to evaluate the fringe amplitude noise. Because the slope at the peak is nearly 0, it is insensitive to fluctuations in the fringe phase. Significantly, we achieved a signal-to-noise ratio (SNR) of 83.4 at the peak of the fringe. The contribution of the fringe amplitude noise can be obtained as follows [40]:

(1) $$\sigma_{g}=\frac{1}{\mathrm{SNR}}\frac{1}{k_{eff}T^{2}},$$

where *k_eff_* is the effective wave vector of the Raman laser, and *T* is the free-evolution time. The value of *σ_g_* is 6.8 μGal/shot, which is mainly due to the combined effects of factors such as laser frequency fluctuations, laser power fluctuations, and atomic number fluctuations.

### 4.2. Phase Noise of the Raman Laser

**Figure 7:**
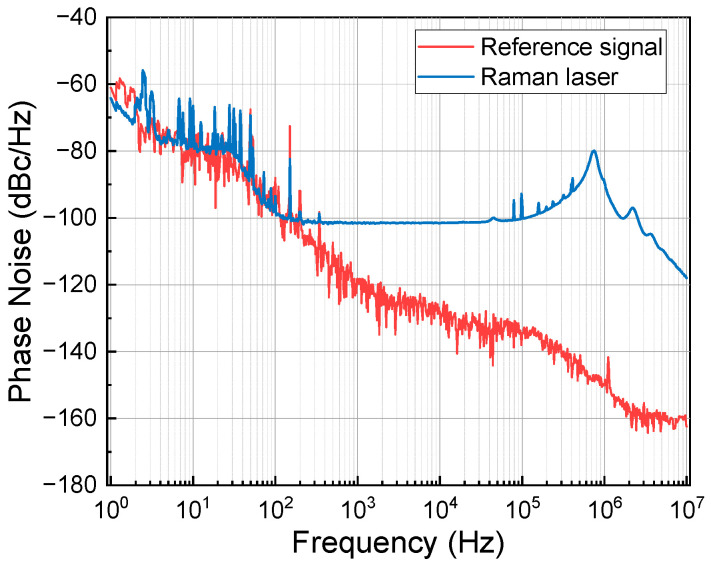
PSD of the phase noise of the Raman laser and reference signal.

An OPLL is used to lock the Raman laser phase, and the details of this process can be found in reference [41]. We used the Keysight E5052B signal analyzer to measure the phase noise of the beat-note signal and the reference signal. The phase noise power spectrum density (PSD) is shown as the blue curve in Figure 7. In a frequency of less than 200 Hz, the phase noise of the Raman laser is limited by the phase noise of the reference signal, as shown as red curve in Figure 7, and the phase noise is better than −100 dBc/Hz in the range of 200 Hz to 100 kHz. The phase noise can be evaluated using [42]

(2) $$\sigma_{\phi }^{2}=\int_{0}^{\infty }{\left|H_{\phi }\left(\omega \right)\right|}^{2}S_{\phi }\left(\omega \right)d\omega ,$$

where $S_{\phi }(\omega )$ is the power density spectrum of the phase noise, as shown as the blue curve in Figure 7. $H_{\phi }(\omega )$ is the phase noise transfer function, and its expression is

(3) $$H_{\phi }\left(2\pi f\right)=i\frac{4}{1-{\left(4\tau f\right)}^{2}}\sin\left(\pi \left(T+2\tau \right)g\right)\cdot [\sin\left(\pi Tf\right)+4\tau f\cos\left(\pi \left(T+2\tau \right)f\right)],$$

where *T* is the free-evolution time and *τ* is the duration time of the *π*/2 pulse. In our system, *T* is 105 ms and *τ* is 18 μs. The contribution of Raman laser phase noise is evaluated to be 2.7 μGal/shot.

### 4.3. Vibration Noise

**Figure 8:**
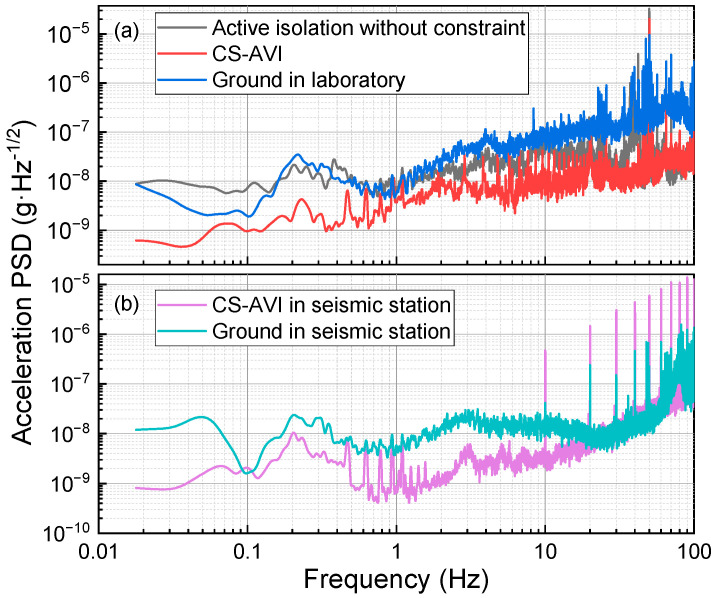
PSD of the vibration noise. (**a**) The vibration noise sampled in the laboratory. (**b**) The vibration noise sampled in the seismic station.

Figure 8a illustrates the PSD of residual vibration noise in the vertical direction of the retroreflector. This measurement was conducted in the laboratory under three different conditions. The blue curve indicates the ground vibration noise in the laboratory. The red and black curves represent the residual vibration noise in the active isolation situation with and without the constraint structure, respectively. When the active vibration isolator is implemented without the constraint structure, the vibration noise is significantly reduced in frequencies above 2 Hz, compared to the ground vibration case. This reduction is attributable to the combined effects of passive and active vibration isolation. However, below 2 Hz, the active isolation without the constraint structure does not attenuate the vibration noise. In fact, the vibration noise is amplified within the frequency range of 0.02–0.15 Hz. This might be due to the rough adjustment of the horizontal level of the passive vibration isolation platform. Consequently, cross-coupling between the horizontal and vertical vibrations may lead to performance degradation of the active isolator within the low-frequency band. However, when the constraint structure is introduced, the cross-coupling between the horizontal and vertical vibrations is suppressed. The red curve in Figure 8a illustrates that under the condition of a constraint structure, the vibration noise is further suppressed. Below 0.5 Hz, the reduction in vibration noise on the red curve is about ten times that of the black curve. Additionally, the vibration noise from 0.5 to 30 Hz is also noticeably attenuated.

The limitation due to ground vibration noise in the laboratory is evaluated to be 62.9 μGal/shot. The primary sources of noise are distributed in the frequency ranges of 0.15–0.4 Hz and 1–5 Hz. Vibration noise above 10 Hz exhibits a higher power characteristic, but it is attenuated in this frequency band due to the significant attenuation effect of the vibrational noise transfer function of the atomic gravimeter at T = 105 ms. The limitation is evaluated to be 33.5 μGal/shot in the case without the constraint structure, whereas the noise reduction mainly stems from the attenuation of vibrations within the 2–10 Hz range. Furthermore, the limitation with the constrained structure is evaluated to be 14.2 μGal/shot, attributed to the attenuation of all vibrations below 10 Hz. This isolation system reduced the ground vibration noise by a factor of 4.4 in the laboratory.

Figure 8b gives the PSD of vibration noise in the transportation test. The green curve shows the vibration noise on the ground of the seismic station, and the effect of vibration noise in this condition is evaluated to be 24.7 μGal/shot. The purple curve shows that the limitation of vibration noise is reduced to 8.7 μGal/shot by CS-AVI. This means that the isolation system still attenuated the ground vibration by a factor of 2.8 even in a quiet seismic station.

### 4.4. Discussion

Based on the above noise analysis, the contributions of amplitude noise of the fringe and phase noise of the Raman laser are determined to be 6.8 μGal/shot and 2.7 μGal/shot, respectively. In comparison to these two types of noise, the ground vibration noise is the primary noise source in the atomic gravimeter, as its contribution surpasses the former two by an order of magnitude. We also took into account other types of noise, such as magnetic field noise and intensity noise of the Raman laser. However, their contributions to the overall noise were significantly smaller compared to the three types of noise that are analyzed here.

In laboratory settings, the impact of vibration noise is reduced to 14.2 μGal/shot through CS-AVI, and the total noise of the gravimeter is significantly reduced to 16.0 μGal/shot, which is considerably smaller than the ground vibration noise limitation of 62.9 μGal/shot. In the transportation test, the overall system noise can be reduced to 11.4 μGal/shot with the implementation of CS-AVI. This is in accordance with the actual result of 10.8 μGal/Hz^1/2^, although there are measurement errors that make our evaluated value slightly larger. This result indicates that other types of noise remain stable after transportation, which highlights the reliability of the gravimeter in transportation.

It should be noted that, in laboratory settings, there is a greater discrepancy between the evaluated system noise and the actual sensitivity compared to this discrepancy in the seismic station. There may be two possible reasons for this problem. First, as shown in Figure 2, the mutual independence among the mirror, retroreflector, and seismometer may mean that the vibration noise measured with the seismometer cannot fully reflect the vibration of the mirror in the retroreflector. Second, the measured vibration noise only represents the in-loop error signal of the PID control circuit, as there was not sufficient space for us to take two seismometers [32] to compare the vibration noise between the in-loop and out-of-loop signals of the control system. The accurate evaluation of the limitations caused by vibration noise requires further research in the future.

## 5. Conclusions

We have achieved transportable high-precision gravity measurement by simplifying the system’s structure and reducing the system noise, especially the vibration noise. The use of a constraint structure can effectively reduce horizontal and vertical cross-coupling, simplifying the vibration isolator structure to make it suitable for transportation measurements. The gravimeter maintains a stable performance during transportation from one site to the other, with a sensitivity of 20.5 μGal/Hz^1/2^ in the laboratory and 10.8 μGal/Hz^1/2^ in the seismic station. In the future, with the completion of a detailed uncertainty evaluation that is currently ongoing for NIM-AGRb2, the atomic gravimeter could be applied as a transportable quantum calibration platform for other gravimeters. The accuracy of the calibration platform can be ensured by tracing the unit of gravitational acceleration to the units of seconds [43] and meters in the international system of units.

## Data Availability

The data in this study are available from the authors upon reasonable request.
